# The Association between Fluoride and Bone Mineral Density in US Children and Adolescents: A Pilot Study

**DOI:** 10.3390/nu16172948

**Published:** 2024-09-02

**Authors:** Haichen Kong, Zihao He, Hui Li, Dan Xing, Jianhao Lin

**Affiliations:** Arthritis Clinic and Research Center, Peking University People’s Hospital, Peking University, Beijing 100044, China; konghaichen@hsc.pku.edu.cn (H.K.); 1710301236@pku.edu.cn (Z.H.); doc_lihui@pku.edu.cn (H.L.); linjianhao@pkuph.edu.cn (J.L.)

**Keywords:** fluoride, bone mineral density, children, adolescents, NHANES

## Abstract

The aim of this study was to examine the association between fluoride exposure and bone mineral density (BMD) in children and adolescents. We used data from the National Health and Nutrition Examination Survey (NHANES) 2015–2016. The fluoride concentrations in the water samples, plasma samples, and urine samples were measured electrometrically using an ion-specific electrode. Total body less head BMD (TBLH BMD) was measured using dual-energy X-ray absorptiometry (DXA). Weighted generalized linear regression models and restricted cubic splines (RCS) regression models were used to analyze the relationships between the three types of fluoride exposure and TBLH BMD. We performed subgroup analyses stratified by sex. A total of 1413 US children and adolescents were included in our study. In our linear regression models, we found inverse associations between fluoride concentrations in water and plasma and TBLH BMD. Additionally, we discovered a non-linear association between fluoride concentrations in water and plasma and TBLH BMD. No significant association or non-linear relationship was found between urine fluoride levels and TBLH BMD. This nationally representative sample study provides valuable insight into the intricate connection between fluoride exposure and skeletal health in children and adolescents.

## 1. Introduction

Bone mineral density (BMD) serves as an indicator of both bone mass and bone strength, and a decrease in BMD has garnered significant attention due to its association with an increased risk of fracture [1]. Attention to BMD in children and adolescents is imperative, as it not only impacts current skeletal health but also significantly influences future quality of life [2,3,4]. In recent years, several studies have investigated the correlation between trace elements and BMD in children and adolescents [5,6].

Fluoride is a trace element that may be found in all water supplies on Earth, with varying amounts and proportions [7]. The concentration of fluoride in groundwater exhibits significant global variation, with some regions exceeding a value of more than 10 mg/L [8]. Some nations add fluoride to drinking water at controlled concentrations as a preventive measure against dental caries [9]. In the United States (US), the implementation of community water fluoridation policies dates back to 1945, with the current water concentration set at 0.7 mg/L [10,11].

Many studies have demonstrated that fluoride exposure can affect bone metabolism, leading to manifestations such as osteoporosis, osteosclerosis, and skeletal abnormalities [12,13]. However, there is no consensus on the effect of fluoride on BMD. Emilie et al. reported a positive correlation between fluoride and BMD in postmenopausal women [14], while another study reached contrasting conclusions [15], and still others found no significant association between fluoride and BMD [16,17]. Additionally, the study population in most articles consisted of elderly women, and there remains a dearth of research investigating the potential association between fluoride exposure and BMD in children and adolescents.

In the present study, we explored the association between fluoride exposure and BMD using a nationally representative sample of children and adolescents in the US. Various indicators of fluoride exposure were incorporated, including drinking water fluoride levels, plasma fluoride concentrations, and urine fluoride concentrations. Both linear and non-linear analyses were performed to comprehensively elucidate the relationship between fluoride exposure and BMD.

## 2. Materials and Methods

### 2.1. Study Design and Participant

This study conducted a cross-sectional analysis using data from the National Health and Nutrition Examination Survey (NHANES), a survey designed to evaluate the health and nutritional status of individuals in the US. The survey incorporated data obtained from questionnaires, laboratory tests, and physical examinations.

**Figure 1:**
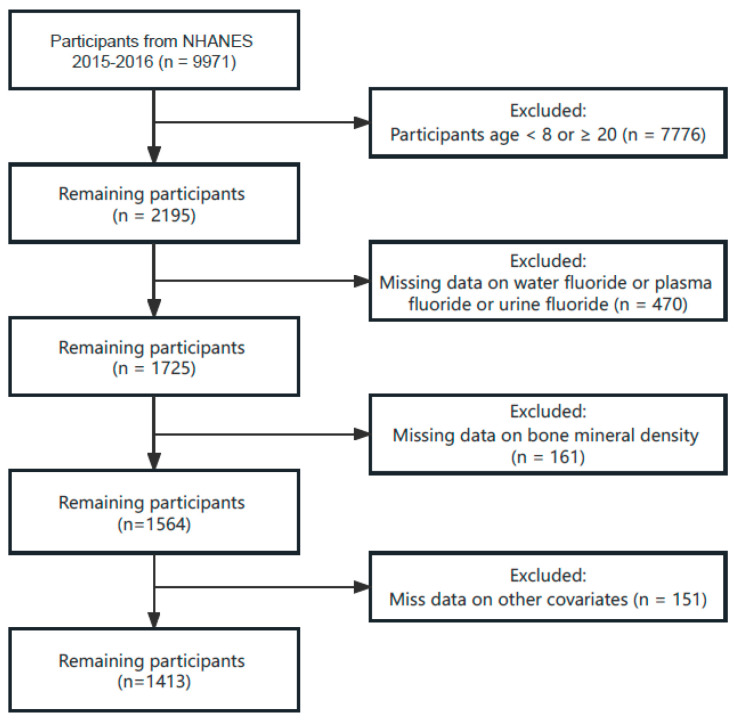
Flow chart of the study participants from NHANES 2015–2016.

The data utilized in this study were obtained from the 2015–2016 cycle of the NHANES and included information on BMD and fluoride levels among individuals aged 8 to 19 years. The inclusion criteria included participants who (1) were aged 8 to 19 years, (2) had data on water fluoride, plasma fluoride, and urine fluoride, (3) had data on BMD, and (4) had data on other covariates. Participants who did not meet the above criteria were excluded. A total of 9971 participants were included in the 2015–2016 cycles of the NHANES. After screening, 1413 participants were selected for the study (Figure 1). The study was approved by the National Center for Health Statistics Research Ethics Review Board, and all adult participants provided written informed consent.

### 2.2. Measurement of Fluoride

The measurement of fluoride concentration in water serves as an indicator for assessing fluoride exposure, while the quantification of fluoride levels in plasma and urine is employed as a biomarker of exposure. Fluoride concentrations in the water samples, plasma samples, and urine samples were measured electrometrically using an ion-specific electrode. Urine samples for fluoride concentration testing were obtained from spot urine collected in the mobile examination center (MEC), rather than from 24-h urine collection. The lower limit of detection (LLOD) for fluoride in plasma water was 0.25 μmol/L, whereas the LLODs for fluoride in water and urine were 0.10 mg/L and 0.144 mg/L, respectively. The value for samples with analysis results below the lower limit of detection was set as LLOD divided by the square root of 2. More detailed descriptions are available in the Laboratory Method Files sections of the NHANES website [18,19,20].

### 2.3. Examination of BMD

BMD (g/cm^2^) was measured using dual-energy X-ray absorptiometry (DXA) examinations, which provides bone measurements for both arms, both legs, the trunk, and the head. General and site-specific BMD data are available on the NHANES website [21]. In the present study, we selected the total body less head BMD (TBLH BMD) as the outcome measurement. The rationale for selecting TBLH BMD over total BMD lies in the fact that the head constitutes a large proportion of the bone mass but exhibits minimal changes related to age and disease. Hence, the exclusion of the head can provide a more accurate representation of overall bone mass alterations [22].

### 2.4. Covariates

The covariates considered in this study included age, sex, race, poverty income ratio (PIR), body mass index (BMI), milk product consumption, and physical activity. In this article, the term ‘children’ refers to individuals aged 8–11 years old, while ‘adolescents’ encompasses those aged 12–19 years old. BMI was calculated by dividing weight (kg) by the square of height (m^2^). The interviewers inquired about the subjects’ frequency of milk product consumption over the past 30 days and collected data through structured questionnaires. The consumption patterns were categorized into three groups: <1 times/week, 1–6 times/week, and >6 times/week. Physical activity in a typical week was categorized into 3 levels: <2 days/week, 2–4 days/week, and >4 days/week. The physical activity level of children was assessed using label ‘days physically active at least 60 min (PAQ706)’, which refers to engaging in any form of physical activity that elevates heart rate and induces labored breathing for at least 60 min per day. The physical activity level of the adolescents was assessed using the label ‘moderate recreational activities (PAQ665)’, which refers to engaging in any moderate-intensity activity that increases heart rate or breath for at least 10 min continuously. More information about milk product consumption and physical activity can be found on the NHANES website’s Questionnaire Instruments Files sections [23,24].

### 2.5. Statistical Analysis

This study followed the NHANES complex sampling survey procedure and used the complex sampling weights provided by the NHANES analytic guidelines. Weighted data were used for indicator analysis to produce nationally representative estimates. We used Student’s *t*-test to compare the mean values of continuous variables between males and females. Chi-square tests were used to compare the percentages of categorical variables between males and females. A weighted generalized linear regression model was used to analyze the relationship between plasma fluoride levels (quartile group) and TBLH BMD, as well as between water fluoride levels and urine fluoride levels. Model 1 was adjusted for age, sex, and race. Model 2 was further adjusted for BMI, PIR, milk product consumption, and physical activity. The weighted β values and 95% confidence intervals (CIs) were calculated. To better demonstrate the dose-response relationships between fluoride levels and TBLH BMD, restricted cubic splines (RCS) with four knots located at the 5th, 35th, 65th, and 95th percentiles of the water fluoride, plasma fluoride, and urine plasma distributions were used, and the covariates adjusted in the RCS regression models were the same as those in linear regression Model 2. Subgroup analysis based on sex was performed with non-linear regression models. Spearman correlation coefficients were calculated between the plasma fluoride concentration and the water fluoride concentration, as well as between the urine fluoride concentration and the water fluoride concentration and between the plasma fluoride concentration and the urine fluoride concentration. A two-sided *p* value < 0.05 was considered to indicate statistical significance. All analyses were performed using R statistical software, version 4.4.0.

## 3. Results

### 3.1. Baseline Characteristics

Table 1 presents the characteristics of the participants in this study. A total of 1413 participants aged ≥ 8 and <20 years old were included. The population consisted of 728 males and 685 females. The average value (mean ± SD) of TBLH BMD was 0.84 ± 0.15 g/cm^2^. The average values (mean ± SD) of the water fluoride concentration, plasma fluoride concentration, and urine fluoride concentration were 0.44 ± 0.35 mg/L, 0.34 ± 0.21 μmol/L, and 0.619 ± 0.510 mg/L, respectively. The differences in the distributions of age, race, physical activity, poverty income ratio, TBLH BMD, and water fluoride concentration between males and females were not statistically significant. However, the differences in the distributions of milk product consumption, BMI, plasma fluoride concentration, and urine fluoride concentration between males and females were statistically significant. Compared with females, males exhibit greater consumption of milk products and a lower BMI. Moreover, both plasma and urine fluoride concentrations were greater in males than in females.

### 3.2. Associations between Fluoride Exposure and TBLH BMD among US Children and Adolescents

Table 2 shows the weighted generalized linear regression model of the concentrations of fluoride exposure and TBLH BMD. In the partially adjusted model (Model 1), we observed inverse associations between the concentrations of plasma fluoride and water fluoride and TBLH BMD (all *p* values for trend < 0.01). The associations persisted when further adjusted for BMI, PIR, physical activity, and milk product consumption (Model 2). However, there was no significant correlation observed between the concentrations of urine fluoride and the TBLH BMD in either Model 1 or Model 2.

**Figure 2:**
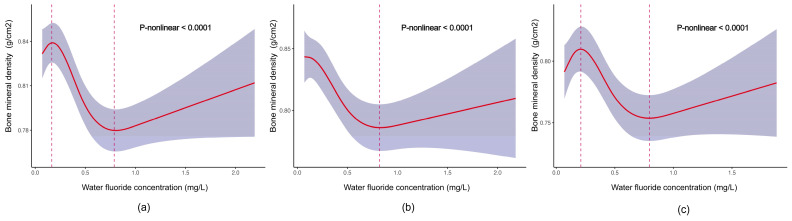
Dose–response relationships between concentrations of water fluoride and TBHL BMD. (**a**) Association between concentrations of water fluoride and TBHL BMD in the whole population; the model was adjusted for age, gender, race, Body Mass Index, poverty income ratio, physical activity, and milk product consumption. (**b**,**c**) Association between concentrations of water fluoride and TBHL BMD in males and females, respectively; the model was adjusted for age, race, Body Mass Index, poverty income ratio, physical activity, and milk product consumption. The red solid lines with their upper and lower ranges represent the estimated regression coefficient Beta and the 95% confidence interval. The red dotted lines correspond to the fluoride concentration at the curve’s inflection point.

The dose-response relationships between the concentrations of water fluoride, plasma fluoride, and urine fluoride and the TBHL BMD were further explored with an RCS graph. Figure 2 shows that there was a significant nonlinear trend between the concentrations of water fluoride and the TBHL BMD in the whole population, in males, and in females (all *p* for trend < 0.0001). In the whole population, the association between water fluoride and TBHL BMD exhibited a positive correlation trend from the lowest level, 0.07 mg/L, to 0.18 mg/L, followed by a subsequent negative correlation, and ultimately reverted to a positive correlation after 0.81 mg/L (Figure 2a). A similar curve is also shown for females in Figure 2c. In males, the association between water fluoride and TBHL BMD was negative before 0.83 mg/L and then became positive (Figure 2b).

**Figure 3:**
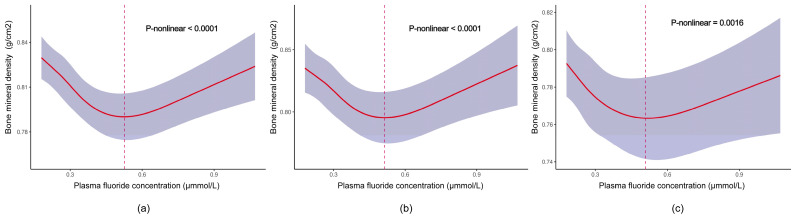
Dose–response relationships between concentrations of plasma fluoride and TBHL BMD. (**a**) Association between concentrations of plasma fluoride and TBHL BMD in the whole population; the model was adjusted for age, gender, race, Body Mass Index, poverty income ratio, physical activity, and milk product consumption. (**b**,**c**) Association between concentrations of plasma fluoride and TBHL BMD in males and females, respectively; the model was adjusted for age, race, Body Mass Index, poverty income ratio, physical activity, and milk product consumption. The red solid lines with their upper and lower ranges represent the estimated regression coefficient Beta and the 95% confidence interval. The red dotted lines correspond to the fluoride concentration at the curve’s inflection point.

Figure 3 shows that there was a significant nonlinear trend between the concentrations of plasma fluoride and TBHL BMD in the whole population, in males, and in females (all *p* for trend < 0.002). In the whole population, the association between plasma fluoride and TBHL BMD exhibited a negative correlation trend from the lowest level, 0.18 μmmol/L, to 0.52 μmmol/L, followed by a subsequent positive correlation (Figure 3a). A similar curve was also found for males (Figure 3b) and for females (Figure 3c).

**Figure 4:**
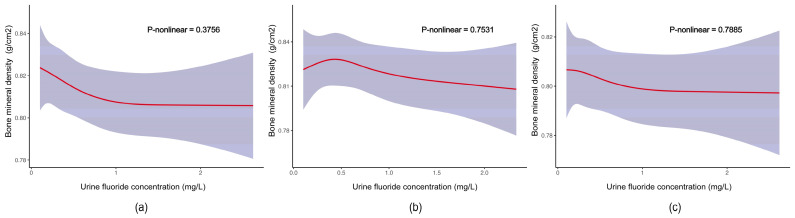
Dose–response relationships between concentrations of urine fluoride and TBHL BMD. (**a**) Association between concentrations of urine fluoride and TBHL BMD in the whole population; the model was adjusted for age, gender, race, Body Mass Index, poverty income ratio, physical activity, and milk product consumption. (**b**,**c**) Association between concentrations of urine fluoride and TBHL BMD in males and females, respectively; the model was adjusted for age, race, Body Mass Index, poverty income ratio, physical activity, and milk product consumption. The red solid lines with their upper and lower ranges represent the estimated regression coefficient Beta and the 95% confidence interval.

Figure 4 shows that there was no significant nonlinear trend between the concentrations of urine fluoride and TBHL BMD in the whole population (*p* for trend = 0.3756), in males (*p* for trend = 0.7531), and in females (*p* for trend = 0.7885). 

### 3.3. Correlation between Concentrations of Different Fluoride Exposure

**Figure 5:**
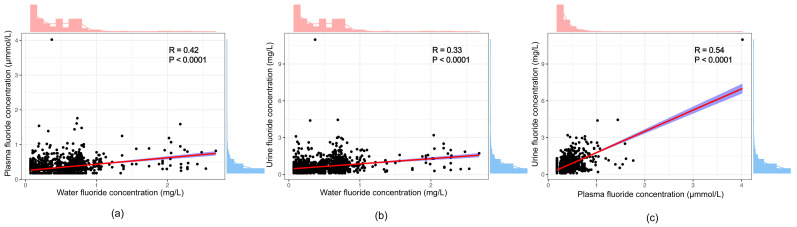
Correlation between concentrations of different fluoride. (**a**) Correlation between concentration of water fluoride and concentration of plasma fluoride. (**b**) Correlation between concentration of water fluoride and concentration of urine fluoride. (**c**) Correlation between concentration of plasma fluoride and concentration of urine fluoride. The red solid lines represent the fitted curve, while the blue shaded areas surrounding them denote the 95% confidence interval for the regression fit. Density plots along the periphery, either red or blue, represent the distribution of data points.

Figure 5a shows that the correlation between the concentration of water fluoride and the concentration of plasma fluoride was positive according to Spearman analysis (*p* < 0.0001, *R* = 0.42). A similar correlation was also found between the concentration of water fluoride and the concentration of urine fluoride, as shown in Figure 5b (*p* < 0.0001, *R* = 0.33), and between the concentration of plasma fluoride and the concentration of urine fluoride, as shown in Figure 5c (*p* < 0.0001, *R* = 0.54).

## 4. Discussion

In this cross-sectional study of a nationally representative sample of US children and adolescents aged 8 to 19 years, we found negative associations between water fluoride and plasma fluoride and BMD via linear analysis. In addition, non-linear dose-response associations were found between the concentration of water fluoride and the concentration of plasma fluoride and BMD in the whole population. Specifically, the correlation between the concentration of water fluoride and the BMD was generally negative when the concentration was below 0.81 mg/L but became positive thereafter. Similarly, a negative correlation between the concentration of plasma fluoride and BMD was observed when the concentration was below 0.52, followed by a positive correlation.

Several studies have assessed the correlation between fluoride exposure and BMD; however, the results are inconclusive. Emilie et al. observed a positive correlation between the concentrations of water fluoride (0–1 mg/L) and BMD in a cohort of postmenopausal women [14]. In contrast, a cross-sectional study involving 722 women reported that the concentration of urine fluoride is negatively related to BMD [25]. A cross-sectional survey involving 907 Chinese farmers [15] and a cross-sectional study involving 943 Chinese residents [26] also reported a negative association between the concentration of urine fluoride and BMD. In addition, a study that included 248 women aged 46–65 years revealed no significant association between the concentration of water fluoride and lumbar BMD [17]. Taken together, disparities in research design, population, fluoride levels, fluoride exposure indicators, and BMD sites may help to partially explain why various studies have yielded conflicting results.

The effect of fluoride on bone metabolism is complex. Fluoride is known to exhibit a bidirectional effect on bone production and resorption. It can enhance osteogenic activity, leading to osteosclerosis, but it can also promote bone resorption, resulting in osteoporosis [12,27]. Many studies have confirmed that fluoride can promote the proliferation and differentiation of osteoblasts and increase bone mass [28,29,30,31]. In terms of osteoclasts, researchers found an inverted U-shaped correlation between fluoride levels and osteoclast formation in a mouse model [32]. These mechanisms partially explain the observation in this study that an increase in fluoride concentration leads to enhanced osteoclastogenesis, resulting in decreased BMD. Furthermore, upon surpassing a certain threshold, higher fluoride levels were found to suppress osteoclast formation while promoting the proliferation and differentiation of osteoblasts, ultimately leading to increased BMD.

In the current study, we found that the association between fluoride and BMD is influenced by sex. We observed a gradual decrease in BMD among males as the concentration of fluoride in water increased from its lowest value (Figure 2b), whereas there was an initial increase in BMD followed by a subsequent decrease in females (Figure 2c). Previous studies have shown that estrogen has a significant effect on bone metabolism by promoting bone formation and reducing bone resorption [33,34]. Based on the above hypothesis, we speculate that the positive association between BMD and water fluoride observed in females with lower levels of water fluoride is attributable to the influence of estrogen. At low fluoride concentrations, the potential protective effect of estrogen on bone may counterbalance the potentially deleterious impact of fluoride on BMD. With increasing fluoride exposure, these protective effects may become overwhelmed, resulting in a subsequent decline in BMD.

Multiple biomarkers, including urine, plasma, nails, hair, saliva, and dental fluorosis, have been employed for the assessment of fluoride exposure [35,36]. In the present study, we examined the correlation between plasma fluoride concentration and BMD, yielding results consistent with the relationship observed between water fluoride concentration and BMD. However, no significant association was found between urine fluoride levels and BMD. These findings suggest that spot urine samples may not be reliable indicators of fluoride exposure in individuals. Indeed, various factors such as collection timing, hydration level, and bladder retention duration can influence an individual’s spot urine sample fluoride concentration [37]. Additionally, inter-individual variations in urinary flow and creatinine excretion rates contribute to differences in spot urine fluoride levels among individuals [38]. Generally, water and blood concentrations of fluoride remain relatively stable over time, providing a more accurate reflection of chronic fluoride exposure effects on bone health. Conversely, spot urine fluoride levels are influenced by various factors, potentially explaining the lack of a significant association between urine fluoride and BMD in this study. Furthermore, it is worth noting that subjects might have been instructed to consume water during the urine collection process in order to expedite urine production, potentially introducing additional variability between measured urinary fluoride levels and actual fluoride exposure levels.

In this study, we investigated the association between fluoride concentration and BMD while considering potential confounding factors including sex, age, race, BMI, PIR, milk product consumption, and physical activity. It is important to recognize that these covariates may influence both BMD and plasma or urine fluoride levels. For example, racial differences in fluoride metabolism could result in varying urine fluoride levels despite similar exposure [39]. Obesity might alter fluoride metabolism through changes in renal function [40], while dietary calcium from milk products can reduce fluoride absorption [41]. Furthermore, physical activity may potentially increase fluoride absorption and retention, thereby raising systemic fluoride levels [42]. Therefore, more research is required to confirm the relationship between these factors and plasma or urine fluoride levels.

The strengths of this study include its nationally representative sample, large sample size, different measurements of fluoride exposure, and the exploration of both linear and non-linear correlations between fluoride exposure and BMD. However, there are several limitations. (1) This study was a cross-sectional study, and a causal relationship between fluoride exposure and BMD could not be determined. (2) The participants included in this study were US children and adolescents. The association between fluoride exposure and BMD may vary among different ethnic groups or regions. (3) Since this study solely utilized TBLH BMD as the dependent variable, it is crucial to conduct further research to explore the effects of fluoride exposure on BMD in different regions. (4) Fluoride in water is not the only source of human intake of fluoride; other possible sources include the use of topical dental products, fluoride drops, and fluoride tablets. (5) Due to the unavailability of serum calcium measurements for individuals aged below 12 years, we were unable to incorporate serum calcium into the covariate analysis. (6) Self-reported levels of milk consumption were utilized as a dietary indicator; however, it should be noted that this measure may not accurately reflect dietary calcium, vitamin D, and protein levels. Additionally, the recall period of 30 days might introduce inconsistencies with actual consumption patterns.

## 5. Conclusions

This study suggested that fluoride concentrations in water and plasma were inversely associated with TBLH BMD in a nationally representative sample of US children and adolescents aged 8–19 years, with their dose-response relationships exhibiting a non-linear trend. However, no significant linear or non-linear association was found between urine fluoride levels and TBLH BMD, possibly due to the use of spot urine collection, which may not provide stable markers of fluoride exposure. These findings provide valuable insights into the intricate relationship between fluoride exposure and skeletal health in children and adolescents.

## Figures and Tables

**Table 1:** Characteristics of included participants.

Characteristic	Overall	Male	Female	*p* Value
Number of participants, n (weighted %)	1413 (100.0)	728 (51.5)	685 (48.5)	
Age group, n (weighted %)				0.6
Adolescents	862 (68.5)	450 (69.2)	412 (67.7)	
Children	551 (31.5)	278 (30.8)	273 (32.3)	
Race, n (weighted %)				0.8
Hispanic	501 (23.7)	240 (23.3)	261 (24.0)	
Non–Hispanic White and Other	481 (59.4)	256 (60.1)	225 (58.7)	
Non–Hispanic Black	299 (12.4)	160 (12.2)	139 (12.5)	
Non–Hispanic Asian	132 (4.6)	72 (4.4)	60 (4.8)	
Physical activity, n (weighted %)				0.8
<2 days/week	479 (39.7)	257 (40.8)	222 (38.5)	
2–4 days/week	519 (35.8)	258 (35.2)	261 (36.5)	
>4 days/week	415 (24.5)	213 (24.0)	202 (25.0)	
Milk product consumption, n (weighted %)				0.001
<1. times/week	198 (14.6)	74 (10.2)	124 (19.5)	
1–6 times/week	367 (26.8)	184 (25.6)	183 (28.1)	
>6 times/week	848 (58.6)	470 (64.3)	378 (52.4)	
Body Mass Index (kg/m^2^)	22.64 (5.89)	22.26 (5.91)	23.05 (5.85)	0.045
Poverty income ratio (means ± SD)	2.49 (1.56)	2.58 (1.56)	2.39 (1.56)	0.13
Bone mineral density (g/cm^2^, means ± SD)	0.84 (0.15)	0.85 (0.17)	0.83 (0.14)	0.058
Water fluoride (mg/L, means ± SD)	0.44 (0.35)	0.44 (0.36)	0.43 (0.34)	>0.9
Plasma fluoride (μmol/L, means ± SD)	0.34 (0.21)	0.36 (0.20)	0.32 (0.21)	<0.001
Urine fluoride (mg/L, means ± SD)	0.619 (0.510)	0.677 (0.498)	0.555 (0.517)	<0.001

**Table 2:** Associations between Fluoride exposure and TBHL BMD in US Children and Adolescents Aged 8–19 Years, NHANES 2015–2016.

	Crude β (95%CIs)	Model 1 β (95%CIs)	Model 2 β (95%CIs)
Water fluoride (mg/L)			
Q1 (≤0.16)	0 (Ref.)	0 (Ref.)	0 (Ref.)
Q2 (0.17–0.42)	−0.03 (−0.05, −0.02) **	−0.01 (−0.03, 0.00)	−0.02 (−0.04, 0.00)
Q3 (0.43–0.68)	−0.06 (−0.11, −0.02) **	−0.04 (−0.09, 0.00)	−0.04 (−0.10, 0.02)
Q4 (>0.68)	−0.08 (−0.13, −0.03) **	−0.06 (−0.10, −0.01) *	−0.06 (−0.12, 0.00)
*p* for trend	<0.001	<0.001	<0.001
Plasma fluoride (μmmol/L)			
Q1 (≤0.18)	0 (Ref.)	0 (Ref.)	0 (Ref.)
Q2 (0.18–0.29)	−0.02 (−0.05, 0.01)	−0.01 (−0.03, 0.01)	−0.01 (−0.03, 0.01)
Q3 (0.30–0.40)	−0.04 (−0.06, −0.02) **	−0.03 (−0.05, −0.01) *	−0.03 (−0.06, 0.00) *
Q4 (>0.40)	−0.04 (−0.08, 0.00)	−0.03 (−0.06, 0.00) *	−0.03 (−0.07, 0.00)
*p* for trend	<0.001	0.009	0.004
Urine fluoride (mg/L)			
Q1 (≤0.299)	0 (Ref.)	0 (Ref.)	0 (Ref.)
Q2 (0.300–0.496)	0.00 (−0.02, 0.02)	0.00 (−0.02, 0.02)	0.00 (−0.03, 0.02)
Q3 (0.497–0.804)	−0.01 (−0.04, 0.03)	0.00 (−0.02, 0.03)	−0.01 (−0.04, 0.03)
Q4 (>0.804)	−0.02 (−0.05, 0.02)	−0.01 (−0.03, 0.01)	−0.01 (−0.04, 0.01)
*p* for trend	0.7	0.8	0.4

TBHL BMD, total body less head bone mineral density; β, estimates of regression coefficients; CIs, confidence interval; Crude Model is the unadjusted model. Model 1 adjusted for age, gender, and race. Model 2 adjusted for age, gender, race, Body Mass Index, poverty income ratio, physical activity, and milk product consumption. * *p* < 0.05; ** *p* < 0.01.

## Data Availability

All data can be found at https://www.cdc.gov/nchs/nhanes (accessed on 1 June 2024).
